# Department of Chemistry and Toxicology

**Published:** 1893-03

**Authors:** Seth M. Morris

**Affiliations:** Medical Department Texas University, Galveston


### DEPARTMENT OF CHEMISTRY AND TOXICOLOGY.

CONDUCTED BY PROF. SETH M. MORRIS, M. D.,
Medical Department Texas University, Galveston.

DIOZOREACTION OF EHRLICH.—This reaction has been known to physiological chemists for about ten years. When concentrated all urines give it, and, therefore, to obtain relatively sharp indications, it is necessary to operate with diluted urine, while to be successful it is also necessary to observe rigorously the directions for the preparation of the reagents as given below. Albumin and peptones are colored yellow and do not become red even on the addition of the solution of ammonia.

The reagents are as follows:

| | | |
|---|---|---|
| No. 1—Sulphoilic acid | .................... | 1 grain. |
| Hydrochloric acid | ................ | 10 c. c. |
| Water | ........................ | 200 c. c. |
| No. 2—Potassium nitrite | ................ | 5 grain. |
| Water | ...... .................. | 100 c. c. |

To make the test, 125 c. c. (4 oz.) of No. 1 and 5 c. c. (⅙ oz.) of No. 2 are mixed together; equal parts of this mixture and urine placed together in a test tube, then about ⅛ of the volume of ammonia water added and the whole shaken and set aside. At the end of twenty-four hours a characteristic green to violet colored precipitate is produced; the liquid itself usually becoming deep red.

This reaction is given by urine of typhoid fever patients during the first week, and is given by the urine in various pulmonary diseases.

So the reaction is very elastic, relative and not of much value.

---

PTOMAINES FROM PATHOLOGICAL URINE.—M. Griffiths (*Repertoir de Pharmacie*) has succeeded in extracting certain ptomaines from the urine in several infectious diseases.

From the urine in cases of scarletina he has obtained a white crystalline ptomaine, soluble in water, of a feeble alkaline reaction, having the same composition as a ptomaine abstracted from cultures of the micrococcus scarlatinæ. Formula ($C_5H_{12}NO_4$).

He has also extracted from the urine of patients from diphtheria, a white crystalline ptomaine, with the formula $C_{14}H_{17}N_2O_6$, identical with that which has been extracted from cultures of the bacillus of Klebs and Loeffler. From the urine of patients with mumps he has obtained a ptomaine, crystallizing in white prismatic needles, with the formula $C_6H_{13}N_3O_4$, corresponding to the constitution of a probable glycocyamine, and which is transformed by oxidation, first, into creatin and then into methyl-guanidine. It is very toxic; administered to a cat, producing nervous excitation, arrest of the salivary secretions, coma and death. These three ptomaines are not found in normal urine and are produced evidently under the influence of the specific microbes of the diseases mentioned.

ANTIDOTE TO HYDROGANIC ACID.—According to Prof. Kobert (*Pearm. Zeit. f. Russl.*) hydrogen peroxide is an antidote to hydroganic acid. His experiments show that the poison may be daily administered to dogs for weeks in succession, without injury, if followed by large doses of hydrogen peroxide internally and subcutaneously as soon as the toxic symptoms appear, the antidote being administered until all odor of the acid disappears from the breath. The hydroganic acid is converted into oxamide.

